# Intake of Phthalate-Tainted Foods Alters Thyroid Functions in Taiwanese Children

**DOI:** 10.1371/journal.pone.0055005

**Published:** 2013-01-30

**Authors:** Ming-Tsang Wu, Chia-Fang Wu, Bai-Hsiun Chen, Eric K. Chen, Yi-Ling Chen, Jentaie Shiea, Wei-Te Lee, Mei-Chyn Chao, Jiunn-Ren Wu

**Affiliations:** 1 Department of Public Health, College of Health Sciences, Kaohsiung Medical University, Kaohsiung, Taiwan; 2 Department of Family Medicine, Kaohsiung Medical University Hospital, Kaohsiung, Taiwan; 3 Center of Environmental and Occupational Medicine, Kaohsiung Municipal Hsiao-Kang Hospital, Kaohsiung, Taiwan; 4 Department of Pediatrics, Kaohsiung Medical University Hospital, Kaohsiung Medical University, Kaohsiung, Taiwan; 5 Superintendant’s Office, Kaohsiung Medical University Hospital, Kaohsiung Medical University, Kaohsiung, Taiwan; 6 Center for General Education, Kaohsiung Medical University, Kaohsiung, Taiwan; 7 Department of Nuclear Medicine, Kaohsiung Medical University Hospital, Kaohsiung Medical University, Kaohsiung, Taiwan; 8 Department of Chemistry, National Sun Yat-Sen University, Kaohsiung, Taiwan; 9 Department of Pediatrics, Kaohsiung Municipal Hsiao-Kang Hospital, Kaohsiung Medical University, Kaohsiung, Taiwan; Cardiff University, United Kingdom

## Abstract

**Background:**

On April-May, 2011, two Taiwan chemical companies were found to have intentionally added phthalates, Di-(2-ethylhexyl) phthalate (DEHP) and/or Di-isononyl phthalate, as a substitute of emulsifier to many foodstuffs. This study aimed to investigate whether exposure to these foods altered endocrine functions in children aged ≤10 years and, if so, whether those changes could be reversed by stopping exposure.

**Methods:**

One *Phthalates Clinic for Children* was established in southern Taiwan between May 31 and June 17, 2011. All eligible children had their exposure information, blood and/or urine specimens collected. Endocrine functions were assessed in serum. The exposure groups were categorized into three (High, >500 ppm, Low, 1–500 ppm, and No, <1 ppm of DEHP). After six months, some children were followed up for the selected endocrine hormones.

**Results:**

Sixty children were eligible in this study; all were Tanner stage 1 with no pubic hair. Compared to non-exposed group, both high and low exposure groups had significantly lower serum thyroid-stimulating hormone (TSH) levels (P = 0.001 and 0.024). At six months follow-up, serum triiodothyronine (T3) levels was significantly changed (P = 0.034) in high exposure group (n = 13). For serum estradiol (E2), the detectable rate (≥8 pg/mL) decreased from 76.9% (10/13) to 30.8% (4/13) (P = 0.070).

**Conclusions:**

This study shows that serum TSH levels can be altered when children were exposed to high concentrations of phthalate-tainted foodstuffs. Serum E2 and T3 may be partially recovered after stopping exposure.

## Introduction

On April-May, 2011, Taiwan authorities discovered that two large local perfumery-chemical companies were intentionally adding phthalates as a substitute of emulsifier (clouding agent) to a variety of foodstuffs [1]. This scandal spread to the whole island and more than 900 foodstuffs were contaminated by two phthalate chemicals, including DEHP (Di-(2-ethylhexyl)phthalate) and/or DINP (Di-isononyl phthalate). Some food products were also being exported to 22 other countries and areas, including Asian countries, the European Union, and the USA [1], [2].

According to the official announcement by the Taiwan Department of Health (TDOH), five food categories were potentially contaminated by DEHP and/or DINP, including (I) Sports drinks, (II) Fruit beverages, (III) Tea drinks, (IV) Fruit jam, nectar, or jelly, and (V) Health food or supplements in tablet, capsule or powder form (TFDA, 2012). Among them, the category of (V) food items, heavily contaminated by DEHP and/or DINP, were regularly taken by infants and children as nutrient supplements or probiotics.

Phthalates such as DEHP and DINP are commonly added to plastics such as polyvinyl chloride for increased flexibility. They are considered to disrupt endocrine function and adversely affect sex and thyroid hormones, the reproductive function, and neurodevelopment [3], [4], [5]. Since this is the first incident where businessmen had intentionally added these two plasticizers to the nutrient supplements for children, we established one *Phthalates Clinic for Children* (PCC) at Kaohsiung Medical University Hospital (KMUH) to investigate whether exposure to these foods altered endocrine functions in children aged ≤10 years (pre-puberty) and, if so, whether those changes could be reversed by stopping exposure.

## Materials and Methods

### Study Subjects

KMUH is a medical center located in Kaohsiung metropolitan of southern Taiwan. This special PCC in KMUH started to screen children who were potentially exposed to phthalates-tainted foodstuffs in May 31, 2011. This study was approved by the Institutional Review Board of KMUH. All children who visited this special clinic and whose parents were willing to participate in this study had their exposure information, blood and urine specimens collected.

### Exposure Information on Phthalates-tainted Foodstuffs

The parents of eligible children were comprehensively interviewed by two trained interviewers to collect the exposure information of intake of phthalates-tainted food items. Between May 27 and July 20, 2011, TFDA published the names of phthalates-tainted food items (DEHP or DINP≥1 ppm) and their makers almost every day on the official website of TDOH for the public [1], so the majority of parents knew well what tainted food items their children were potentially exposed to. In addition, these two interviewers updated the new contaminated food items almost every day from the official website of Taiwan Food and Drugs Administration (TFDA). If the interviewers and the parents identified that the children were suspiciously exposed to any contaminated food item, detailed exposure information, including the exposure unit (bottle, cup, sachet, or tablet), frequency (times per day, week or month), and duration of intake (weeks, months or years), was recorded. The exposure unit could be converted to an amount of exposure based on the exposure information from TFDA [1]. Since the Bureau of Health of Kaohsiung City also provided us with the phthalates concentrations of 1,884 suspected affected food items [1], we were able to construct total daily intake dose (TDI, mg/kg body weight (bw)/day) of more than half of the studied children on the basis of the exposure amount (mg per time) and frequency (times per day) divided by body weight (kg) of each child.

### Measurement of Endocrine Hormones in Serum

After the physical examination of pubertal stage (Tanner stage) by pediatricians, blood samples of the children were collected by phlebotomy and immediately centrifuged at 4°C for 20 min. The supernatant of serum was aliquoted and stored at −80°C until analysis. Urine samples were also collected for routine examination in the clinic immediately and the rest of urine samples were stored in −20°C.

Besides the regular measurement of liver and kidney functions in serum, which include aspartate aminotransferase (AST), alanine aminotransferase (ALT), blood urea nitrogen (BUN), and creatinine, the serum samples were also quantified endocrine profiles by radioimmunoassay (RIA). The endocrine profile included thyroid-stimulating hormone (TSH), thyroxine (T4), free thyroxine (FT4), triiodothyronine (T3), estradiol (E2), testosterone (TT), follicle-stimulating hormone (FSH), and luteinizing hormone (LH). TSH, T4, FT4, and T3 were measured by RIA-gnost^®^ Cisbio Bioassays (Cisbio Bioassays International-CISBIO, Saclay, France), whereas E2, TT, FSH, and LH were measured by Coat-A-Count^®^ kit (*Siemens* Healthcare Diagnostics, Los Angeles, CA, USA). All endocrine profiles were analyzed by RIA in a clinical laboratory of the Department of Nuclear Medicine, KMUH, by one senior laboratory technician (YL Chen) who was blinded to the exposure status of the study children. The clinical laboratory was officially accredited by Taiwan Accreditation Foundation based on the accreditation criteria of ISO 15189∶2007 and the effective period of this certificate (Certificate No. L1905–110705) was between 20 May 2011 and 19 May 2014. The detailed analytical methods and their quality controls have been described previously [6]. The analytical sensitivity of radioimmunoassay was 0.03 µU/mL for TSH, 0.25 µg/dL for T4, 0.05 ng/dL for FT4, 10.0 ng/dL for T3, 8 pg/mL for E2, 4 pg/mL for TT, 0.15 mIU/mL for LH, and 0.06 mIU/mL for FSH. After six months, 22 subjects were followed-up for their serum TSH, T4, FT4, T3, and E2.

In order to verify the quality control of laboratory, we randomly selected 10 study children and asked this senior technician, who was blinded to sample selection origination, to measure TSH levels in plasma and serum by RIA. We found excellent correlation between the two (Spearman correlation coefficient r = 0.988; P<0.0001, Figure S1). We also used the method of enzyme immunoassay (TSH3-Ultra ADVIA Centaur® XP immunoassay Systems, Siemens Healthcare Diagnostics Ltd., Camberley, UK) to measure serum TSH levels and compared that by RIA in serum and still found good correlation (Spearman correlation coefficient r = 0.891; P = 0.001, Figure S2).

### Statistical Analyses

Mean ± standard deviation (SD), median (interquartile range, IQR) or number (frequency) was used to describe the demographic characteristics and clinical findings when appropriate. Since all serum TSH, T4, FT4, and T3 levels in the baseline and at follow-up were above analytical sensitivity and only one measurement of FSH were below analytical sensitivity, TSH, T4, FT4, T3, and FSH levels were treated as continuous variables. For E2, TT, and LH, 41.7%, 65.0%, and 78.3% of measurements were below analytical sensitivity, so these variables were dichotomized based on their analytical sensitivity (8 pg/mL for E2, 4 pg/mL for TT, and 0.15 mIU/mL for LH).

Mann-Whitney U test for continuous variables and Fischer’s exact test for categorical variables were used to compare the differences between exposed and non-exposed groups based on definitions of the affected food by exposure information from TFDA or Bureau of Health of Kaohsiung City in which the concentration of DEHP or DINP≥1 ppm. Since TFDA only published the highest DEHP or DINP concentrations of affected food items to stop people from consuming them and the heavily phthalates-tainted foodstuffs were those mainly from nutrient supplements or probiotics mostly consumed by infants and children [1], we also categorized subjects who were known to be exposed to >500 ppm of DEHP in affected food items as the high exposure group. The low exposure group was those known to be exposed to 1–500 ppm of DEHP in affected food items and those exposed to affected foods, but with unknown DEHP concentration. The sample size was relatively small in this study, so Monte Carlo permutation test with the random permutation number of 10,000 was also used to compare serum endocrine profiles in the exposure group (high or low) with the non-exposed group. False discovery rate (FDR) was used to correct the p-values for two comparisons. The significant serum hormones in the univariable analyses among the exposure groups were further analyzed by multivariable regression analyses after adjusting for age and gender.

For subjects exposed to known DEHP concentrations of foodstuffs, their exposure statuses were also categorized by the recommended tolerable intake dose (TDI) of DEHP from the U.S. Environmental Protection Agency (USEPA, TDI <0.02 mg/kg/day) and European Food Safety Authority (EFSA, TDI <0.05 mg/kg/day) [7], [8]. Kruskal-Wallis test or Fischer’s exact test and trend test were used to compare differences between the three exposed groups (TDI >0.05, ≤0.05 and >0.02, and ≤0.02 and >0.0) and one non-exposed group. Mann-Whitney U test or Fischer’s exact test was used to compare the differences between the three exposed groups *vs.* the non-exposed group. The Spearman correlation coefficient was used to examine the correlation between TDI and selected serum hormone levels with continuous variables.

The differences of serum hormone levels between the baseline and six months follow-up were compared by Wilcoxon signed ranks test or McNemar’s test. The data were analyzed using the SPSS statistical software version 14.0 or STATA statistical software version 10.0.; all *p*-values were two-sided and the significance of *p*-value was <0.05.

## Results

**Figure 1:**
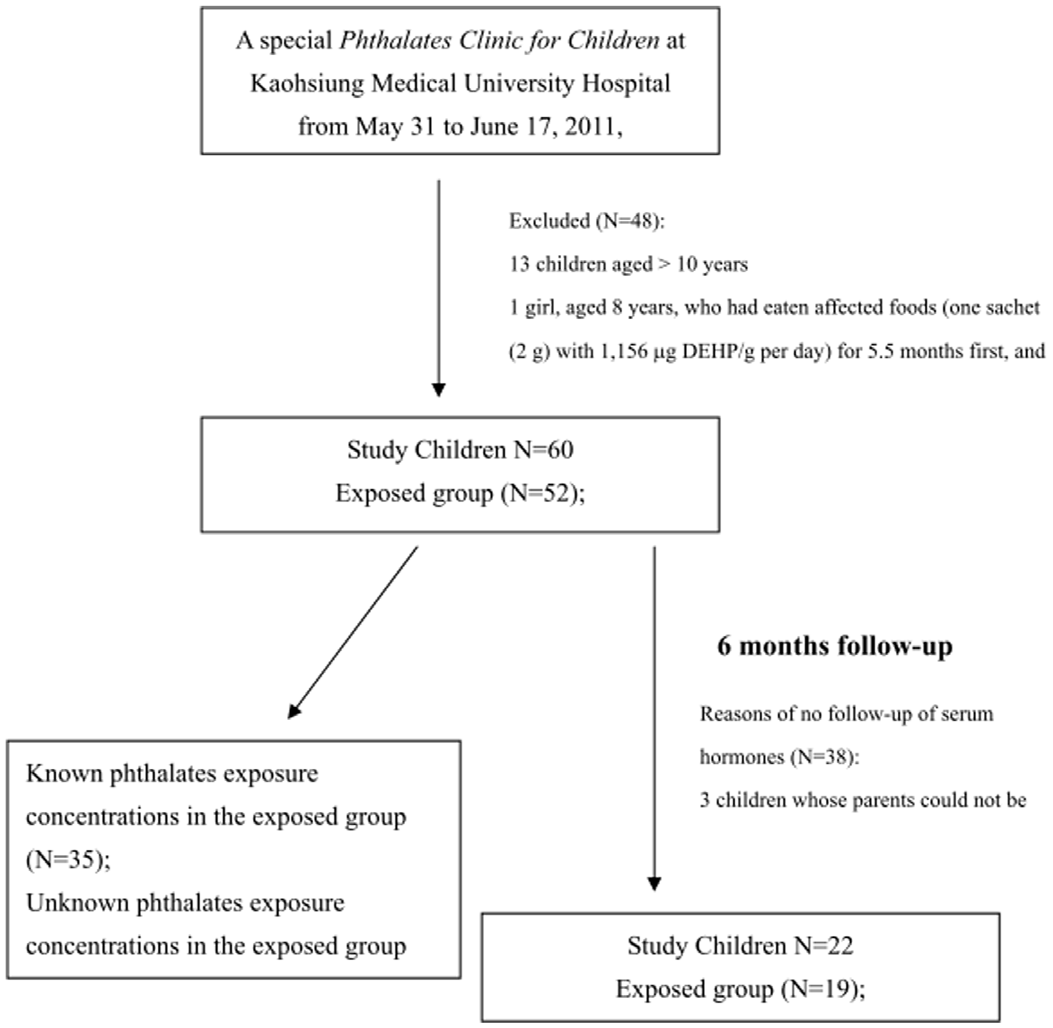
Flow chart of this study (known phthalate concentrations of affected foods were obtained from two sources: the official website of Taiwan Food and Drugs Administration and the Kaohsiung City Bureau of Health (Wu et al., 2012)).

Between May 31 and June 17, 2011, 108 children visited the PCC (Figure 1). Thirteen children whose age was older than 10 years, one girl with a history of hormone (leuprorelin) treatment, and thirty-four whose parents did not sign informed consents were excluded, with 60 children remaining eligible to participate in this study.

All sixty subjects were at Tanner stage 1 without the presence of pubic hair. The mean (± SD) ages (years) were 4.8 (±2.7) and 5.2 (±3.0) in the high and low exposure groups respectively, and 4.5 (±2.6) in 8 non-exposed children (Table 1). Compared to the non-exposed group, we found that both high and low exposure groups had significantly lower serum TSH levels (Mann-Whitney U test, P = 0.011 and 0.047) (Table 1). The significant lower TSH levels were still present when combined high and low exposure groups and compared with the non-exposed group (P = 0.014, Table S1). Using the Monte Carlo permutation test with the random permutation number of 10,000, we still found that the median serum TSH levels in the high and low exposure groups were significantly lower than that in the non-exposure group (Permutation test: FDR_P = 0.009 and 0.047).

**Table 1:** Characteristics and Clinical Findings of Study Children Categorized by Exposure Groups.

	High Exposure	Low exposure	No exposure	
	(>500 ppm)	(1.0–500 ppm)	(<1.0 ppm)	
	N = 29	N = 23	N = 8	P Value^1^
Mean ± SD (Median, IQR) or N (%)				
Age (yrs)	4.8±2.7	5.2±3.0	4.5±2.6	0.820
	(5.0, 2.0–6.0)	(5.0, 3.0–8.0)	(4.0, 3.0–6.0)	
Gender				
Female	10 (34.5)	4 (17.4)	1 (12.5)	0.318
Male	19 (65.5)	19 (82.6)	7 (87.5)	
Height (cm)	106.0±20.3	111.2±22.5	106.2±16.3	0.729
	(110.0, 88.0–123.0)	(111.0, 90.0–133.0)	(107.0, 99.0–124.0)^2^	
Weight (kg)	18.9±8.9	24.8±15.6	18.9±6.0	0.563
	(18.0, 12.5–22.5)	(20.0, 12.5–36.0)	(20.0, 14.5–24.0)^2^	
BMI (kg/m^2^)	16.0±2.3	18.1±4.4	16.4±1.6	0.264
	(15.7, 14.9–16.8)	(16.1, 15.4–20.2)	(16.4, 14.8–17.5)^2^	
Waist circumference (cm)	51.9±8.3	54.2±15.8	55.0±4.4	0.485
	(51.0, 46.0–55.0)	(50.8, 45.5–59.0)	(54.5, 52.0–57.2)^3^	
Hip circumference (cm)	57.9±10.7	60.7±17.5	63.1±5.3	0.338
	(58.0, 50.0–65.0)	(57.0, 51.0–75.0)	(64.0, 60.0–68.0)^3^	
Endocrine findings				
**TSH (µU/mL)** 5	**2.80±1.18**	**3.41±1.23**	**4.73±1.92**	**0.011**
	**(2.94, 2.28–3.21)**	**(3.51, 2.73–4.20)**	**(4.74, 3.71–5.70)**	
FT4 (ng/dL)	1.17±0.17	1.21±0.16	1.23±0.26	0.849
	(1.19, 0.99–1.28)^3^	(1.17, 1.13–1.23)^4^	(1.24, 1.02–1.39)^2^	
T3 (ng/dL)	146.5±21.3	143.9±25.0	147.4±22.5	0.883
	(146.1, 127.3–169.4)^3^	(141.2, 131.0–159.6)^4^	(134.4, 129.8–164.3)^2^	
T4 (µg/dL)	9.07±2.01	8.69±2.15	8.51±1.97	0.713
	(8.94, 7.64–10.80)^3^	(8.19, 7.63–9.93)^4^	(7.83, 7.21–11.11)^2^	
**E2 (pg/mL)** 6				
**<8**	**6 (20.7)**	**15 (65.2)**	**4 (50.0)**	**0.003**
**≥8**	**23 (79.3)**	**8 (34.8)**	**4 (50.0)**	
TT (ng/dL)				
<4	21 (72.4)	14 (63.6)^2^	4 (50.0)	0.481
≥4	8 (27.6)	8 (36.4)	4 (50.0)	
LH (mIU/mL)				
<0.15	23 (79.3)	17 (73.9)	8 (100.0)	0.371
≥0.15	6 (20.7)	6 (26.1)	0	
FSH (mIU/mL)	1.83±1.63	2.03±1.55	1.36±0.89	0.507
	(1.34, 0.82–2.30)	(1.54, 1.10–2.40)	(1.20, 0.74–2.10)	
Biochemical findings				
AST (IU/L)	33.6±8.1	30.0±5.9	30.9±4.3	0.355
	(30.0, 28.0–39.0)	(30.5, 25.0–35.0)^2^	(29.5, 29.0–31.0)	
ALT (IU/L)	16.3±4.8	19.5±11.0	16.5±2.5	0.666
	(15.0, 14.0–17.0)	(16.0, 13.0–21.0)^2^	(17.5, 14.0–18.5)	
BUN (mg/dL)	10.4±3.2	11.3±3.3	12.2±2.2	0.257
	(10.0, 7.2–12.9)	(10.2, 8.8–13.4)	(12.1, 10.1–14.4)	
Creatinine (mg/dL)	0.35±0.10	0.37±0.12	0.33±0.10	0.684
	(0.36, 0.26–0.41)	(0.36, 0.30–0.46)	(0.32, 0.27–0.39)	
Urinalysis findings				
Proteinuria (>1+)	0	2	0	-
Occult blood (>1+)	0	0	0	-
Hematuria (red-cell count,	0	0	0	-
>5/HPF)				
Pyuria (white-cell count,	2	0	0	-
>5/HPF)				

Abbreviations: IQR: Interquartile range; BMI: Body mass index; TSH: Thyroid-stimulating hormone; T4: Thyroxine; FT4: Free thyroxine; T3: Triiodothyronine; E2: Estradiol; LH: Luteinizing hormone; FSH: Follicle-stimulating hormone; AST: Aspartate aminotransferase; ALT: Alanine aminotransferase; BUN: Blood urea nitrogen; HPF: High power field.

1 Kruskal-Wallis test for continuous variables and Fischer’s exact test for category variables.

2 One missing data.

3 Two missing data.

4 Six missing data.

5 Mann-Whitney U test: High exposure group *vs.* no exposure group, P = 0.011; low exposure group *vs.* no exposure group, P = 0.047; trend test, P = 0.001.

6 Fischer’s exact test: High exposure group *vs.* no exposure group, P = 0.174; low exposure group *vs.* no exposure group, P = 0.676; trend test, P = 0.013.

Using the univariable linear regressions, we found the β coefficients (standard error (SE)) of serum TSH levels in the high and low exposure groups was −1.93 (0.52) and −1.32 (0.54) respectively (P = 0.001 and 0.017). After adjusting for age and gender, the β coefficients (SE) in the high and low exposure groups became −1.78 (0.52) and −1.24 (0.53) respectively, which were still statistically significant (P = 0.001 and 0.024).

Although the frequency of detectable serum E2 levels were significantly different among the three exposure groups (P = 0.003), the frequency of detectable serum E2 levels were higher in the high exposure group, but lower in the low exposure group, when compared to the non-exposure group (Table 1). The frequency of detectable serum E2 levels in both high and low exposure groups was not significantly different from the non-exposure group (P = 0.174 and 0.676). The remaining endocrine hormone levels, including T4, FT4, T3, TT, FSH, and LH, were also not significantly different between the three exposure groups. In addition, liver and kidney functions were also similar among the three exposure groups.

Of thirty-five exposed children with information about phthalate exposure concentrations, their exposure concentration (ppm) ranged from 2.74 to 2,108 for DEHP and from <1 to ∼8,713 for DINP (Table 2). Among them, twenty-nine (82.9%) and seventeen (48.6%) of them exceeded TDI of DEHP from USEPA and EFSA respectively.

**Table 2:** Information of Intake-Affected Foods among the 52 Exposed Children.

Concentrations of intake-affected foods^1^	Known	Unknown
	N = 35	N = 17
**Minimum, median, maximum**		
Concentration (ppm)		
DEHP	2.74, 527, 2108	^−2^
DINP	<1, ∼8713, ∼8713	^−2^
Duration (day)	15, 360, 1620	30, 360, 720
Daily intake (mg/kg/day)		
DEHP	0.0028, 0.0498, 0.1874	^−3^
DINP	0, 0.6970, 3.0980	^−3^
**N (%)**		
TDI of DEHP (mg/kg/day)		
>0.02 of U.S. EPA	29 (82.9)	^−3^
>0.05 of EFSA	17 (48.6)	^−3^
TDI of DINP (mg/kg/day)		
>0.15 of EFSA^4^	20 (57.1)	^−3^

Abbreviations: DEHP: Di-(2-ethylhexyl)phthalate; DINP: Di-isononyl phthalate; TDI: Tolerable daily intake; EPA: Environmental Protection Agency; EFSA: European Food Safety Authority.

1 Known phthalates concentrations of affected foods were from two sources: the official website of Taiwan Food and Drugs Administration (TFDA) and Kaohsiung City Bureau of Health.

2 Either DEHP or DINP concentration in affected food ≥1 ppm.

3 No information.

4 No TDI information of DINP from U.S. EPA.

**Figure 2:**
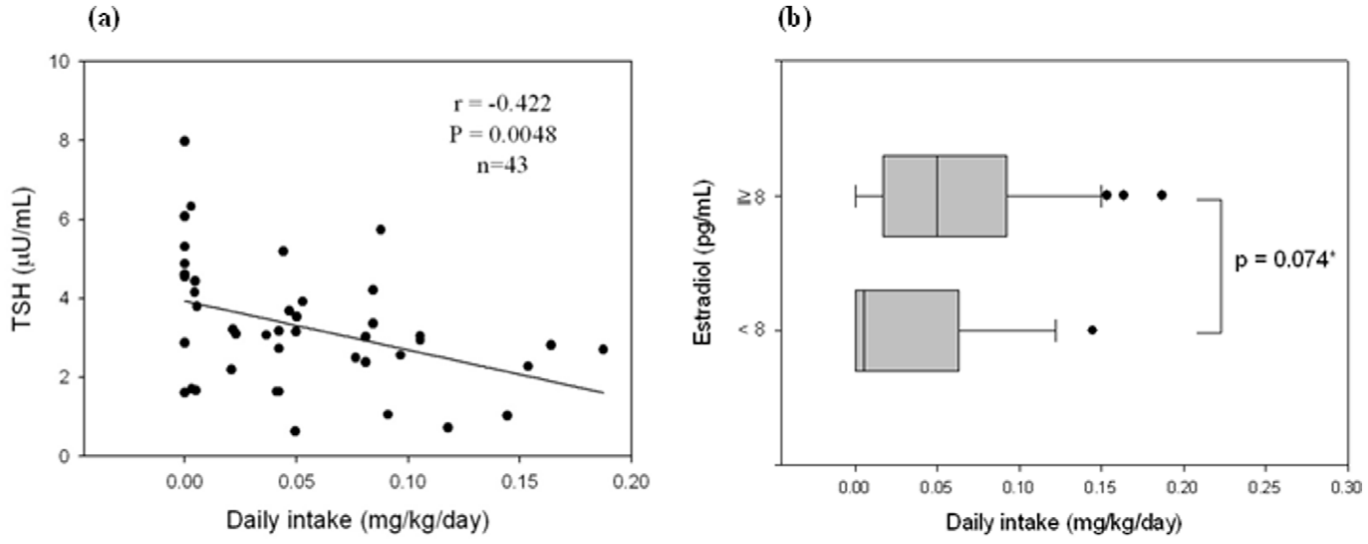
The relationship between daily intake (mg/kg/day) of affected foods and selected endocrine hormones. a) Spearman correlation between daily intake of affected foods and serum thyroid-stimulating hormone (TSH); b) median daily intake dichotomized by analytical sensitivity (8 pg/mL) of serum estradiol (E2) levels (*Mann-Whitney U test).

When categorized by TDIs from USEPA and EFSA, we found that those exposed to >0.05 mg/kg bw/day and exposed to 0.02 and 0.05 mg/kg/day of DEHP had significantly lower serum TSH levels than the non-exposed group (Mann-Whitney U test, P = 0.014 and 0.037, Table 3). Serum TSH levels were negatively associated with daily intake of known contaminated foods (Spearman correlation coefficient r = 0.422, P = 0.0048, Figure 2a). For E2, we found that children with detectable serum E2 levels (≥8 pg/mL) had higher [reaching marginal significance (P = 0.074)] daily intake of DEHP-tainted foods than those with non-detectable serum E2 levels (<8 pg/mL) (median (IQR) daily intake (mg/kg/day): 0.050 (0.021–0.091) *vs.* 0.006 (0–0.044), Figure 2b).

**Table 3:** Serum Thyroid Profiles and Estradiol Levels Categorized by Tolerable Daily Intake (TDI) of U.S. Environmental Protection Agency and European Food Safety Authority among the 35 Exposed Children of Known Concentrations of Affected Foods and 8 Non-exposed Children.

	Exposed groups			Non-exposed		
TDI (mg/kg/day)	>0.05	≤0.05, >0.02	≤0.02, >0	group	P value^1^	P value for trend
	N = 17	N = 12	N = 6	N = 8		
Mean ± SD (Median, IQR) or N (%)						
TSH (µU/mL)	2.81±1.22^4^	2.79±1.16^3^	3.68±1.78^2^	4.73±1.92^2–4^	0.054	0.003
	(2.82, 2.39–3.37)	(3.09, 1.92–3.20)	(3.98, 1.70–4.44)	(4.74, 3.71–5.70)		
T4 (µg/dL)	9.41±2.48^4^	8.58±0.88^3^	9.06±2.59^2^	8.51±1.97^2–4^	0.680	0.362
	(9.69, 7.63–11.13)	(8.81, 7.70–9.41)	(9.66, 7.63–10.19)	(7.83, 7.21–11.11)		
FT4 (ng/dL)	1.16±0.13^4^	1.19±0.23^3^	1.31±0.25^2^	1.23±0.26^2–4^	0.762	0.226
	(1.17, 1.05–1.26)	(1.19, 0.96–1.36)	(1.23, 1.20–1.27)	(1.24, 1.02–1.39)		
T3 (ng/dL)	149.65±20.85^4^	141.96±22.17^3^	137.37±18.89^2^	147.41±22.54^2–4^	0.677	0.590
	(151.36, 130.58–166.87)	(141.94, 124.81–169.39)	(134.03, 131.01–151.19)	(134.43, 129.84–164.28)		
E2 (pg/mL)						
<8	3 (17.6)^4^	3 (25.0)^4^	3 (50.0)^4^	4 (50.0)^2–4^	0.258	0.058
≥8	14 (82.4)	9 (75.0)	3 (50.0)	4 (50.0)		

Abbreviations: IQR: Interquartile range; TSH: Thyroid-stimulating hormone; T4: Thyroxine; FT4: Free thyroxine; T3: triiodothyronine; E2: Estradiol.

1 Kruskal-Wallis H or Fisher’s exact test among the 4 groups.

2 Mann-Whitney U test or Fischer’s exact test: P value = 0.245 for TSH, 0.685 for T4, 0.626 for FT4, 0.570 for T3, and 1.000 for E2, when compared to the non-exposed group.

3 Mann-Whitney U test or Fischer’s exact test: P value = **0.037** for TSH, 0.469 for T4, 0.684 for FT4, 0.751 for T3, and 0.356 for E2, when compared to the non-exposed group.

4 Mann-Whitney U test or Fischer’s exact test: P value = **0.014** for TSH, 0.423 for T4, 0.616 for FT4, 0.947 for T3, and 0.156 for E2, when compared to the non-exposed group.

At six months, the remaining 19 exposed children (another 3 were non-exposed) were found to have significantly lower median T3 levels than at baseline (−16.39 ng/dL, P = 0.019) (Table S2). The significant result of lower median T3 levels was particularly found in the high exposure group (−30.30 ng/dL, n = 13, P = 0.034, Table 4). For serum E2 levels, 10 (76.9%) out of 13 children in the high exposure group at the baseline had detectable serum E2 levels (≥8 pg/mL). After 6 months, the detectable rate decreased to 30.8% (4/13), which was marginally significant (McNemar’s test, P = 0.070). Serum E2 levels did not change significantly in the low exposure group (n = 6) and non-exposure group (n = 3, data not shown).

**Table 4:** Differences of Endocrine Hormone Levels in Serum Between 6 Months Follow-up and Baseline Categorized by Exposure Groups.

Differences	High Exposure		Low exposure		No exposure	
(Levels at 6 months follow-up − baseline)	(>500 ppm)		(1.0–500 ppm)		(<1.0 ppm)	
	N = 13	P Value^1^	N = 6	P Value^1^	N = 3	P Value^1^
Mean ± SD (Median, IQR) or N (%)						
TSH (µU/mL)	0.40±2.02	0.972	−0.17±1.11	0.463	−1.49±0.46	0.109
	(−0.06, −1.17– 0.46)		(−0.24, −1.24 − 1.06)		(−1.72, −1.78 – −0.96)	
Free T4 (ng/dL)	−0.12±0.44	0.722	−0.42±0.51	0.173	−0.84±0.62	0.109
	(0.01, −0.41–0.20)^2^		(−0.49, −0.87– 0.07)		(−1.08, −1.30–0.13)	
**T3 (ng/dL)**	**−22.47±26.35**	**0.034**	−0.74±8.26	0.893	−15.40±58.26	0.593
	**(−30.30, −37.07 –15.90)** 2		(0.19, −7.29–5.71)		(−13.24, −74.71−41.76)	
T4 (µg/dL)	0.78±3.12	0.480	1.30±1.92	0.173	2.97±2.90	0.109
	(1.49, −2.14–3.42)^2^		(1.93, −0.03−2.90)		(3.09, 0.02−5.81)	

Abbreviations: IQR: Interquartile range; TSH: Thyroid-stimulating hormone; T4: Thyroxine; FT4: Free thyroxine; T3: Triiodothyronine.

1 Wilcoxon Signed Ranks Test.

2 One missing data.

## Discussion

This study found that serum TSH can be altered in children exposed to phthalate-tainted foodstuffs and that T3 may be partially recovered after stopping exposure. In addition, USEPA-recommended TDI was found better able to protect this susceptible population than the EFSA TDI, in terms of serum TSH.

This scandal happened around April-May, 2011 and appeared to be winding down in August, 2011 [1]. Although several articles, including ours, have reported the course of this incident and its impact in Taiwan society [1], [2], [9], [10], to our knowledge, this is the first study to examine the adverse effects of this phthalate-tainted foodstuffs incident in children. The highest concentrations of DEHP and DINP-contaminated foodstuffs were found in two popular probiotics, *Bifi DODO* (2,108.0 ppm of DEHP) and *Power-Lac Nutrition Powder* (∼8,713.0 ppm of DINP) [1]. Before this incident, these two products were regularly taken as nutriments by some infants and children in Taiwan. Based on the reference doses (RfD) of DEHP established by EPA and TDI of DEHP of EFSA, which were 0.02 and 0.05 mg/kg bw/day respectively [7], [8], we found that children weighing 20 kg taking one tablet (500 mg) *Bifi DODO* would exceed both the EPA RfD and EFSA TDI for DEHP [1].

According to the experimental studies [11], [12], [13], the disruption of thyroid function by phthalates such as DEHP has been proposed as follows: 1) Affecting the T3 binding to transport proteins; 2) Interacting with the uptake of active T3 in plasma membrane; and 3) Acting as an antagonist at the thyroid receptors, etc. Several human studies have examined the relationship between DEHP metabolites in urine and serum thyroid profiles in adults, adolescents, or children [14], [15], [16]. Meeker and his coworkers firstly studied 408 men and collected their blood and one-spot urine samples when they visited one Fertility Center in Massachusetts, USA, for infertility evaluation [14]. They measured DEHP metabolites, including mono(2-ethylhexyl) phthalate (MEHP), mono-(2-ethyl-5-hydroxylhexyl) phthalate (MEHHP), and mono-(2-ethyl-5-oxohexyl) phthalate (MEOHP), in urine and TSH, FT4, and T3 in serum. They found a significantly inverse association between MEHP and serum FT4 levels, but not TSH and T3, after adjusting for other covariates. In contrast, MEHHP was significantly and positively associated with FT4, but not TSH and T3, in a subgroup of 208 study men. Recently, Meeker & Ferguson studied another 1,346 adults whose age ≥ 20 years and 329 adolescents aged 12–19 years from the 2007–2008 National Health and Nutrition Examination Survey (NHANES) [15]. One-spot urine was also used for measuring DEHP metabolites (MEHP, MEHHP, MEOHP, and mono(2-ethyl-5-carboxypentyl) phthalate (MECPP), whereas TSH, T4, FT4, T3, and thyroglobulin were measured in serum. They found that only MEHHP of DEHP metabolites displayed monotonic dose-dependent decreases in T4, but not other thyroid profiles. In addition, all four DEHP metabolites were not significantly associated with TSH. In adolescents, they observed a significant and positive association between DEHP secondary metabolites and T3 and TSH. In one child study, Boas and his colleagues studied 845 subjects, aged 4–9 years, in Denmark between 2006–7. They measured 12 phthalates metabolites and serum levels of thyroid hormones (TSH, T4, FT4, T3, and free T3 (FT3)) [16]. They found the sum of concentrations of DEHP metabolites (MEHP, MEHHP, MEOHP, and MECPP, corrected by their molecular weights) were negatively associated with FT3, but this significant result was absent when creatinine was corrected. In contrast, they did not find an association between the sum of concentrations of DEHP metabolites and other thyroid hormones, including TSH, T4, FT4, and T3. Although these studies suggest DEHP metabolites may disrupt the thyroid signaling in adults and children, several issues are most concerning. First, the alteration of which particular thyroid hormone by which DEHP metabolite was inconsistent. Second, one-spot urine was used to measure phthalates metabolites, which may not be sufficient to represent long-term exposure, since the considerable inter-day variability of MEHHP over one week was suggested by Preau et al. [17]. Third, how to weigh the toxic effect, in terms of endocrine function of individual DEHP metabolites to become total concentrations of DEHP metabolites is problematic. Boas et al. used the sum of concentrations of DEHP metabolites corrected by their molecular weights [16] and another two studies only examined the individual DEHP metabolite effect [14], [15]. Fourth, all those studies were cross-sectional; the causality of DEHP exposure and thyroid adverse effect was doubtful [18]. Our study may not solve those issues, but we did find serum TSH levels decreased in children exposed to heavily DEHP tainted foods and T3, but not TSH, which may be changed after stopping exposure 6 months later. This result suggests a long-term follow-up of serum TSH levels is necessary to assess their recovery.

Phthalates can also adversely affect sex hormones such as E2 [3], [4], [5], [18]. This study found that children with detectable serum E2 levels (≥8 pg/mL) had marginally and significantly higher daily intake of DEHP-tainted foods than those with non-detectable serum E2 levels (<8 pg/mL) (P = 0.074). After 6 months follow-up, the number of study children with detectable serum E2 levels (≥8 pg/mL) marginally and significantly decreased (P = 0.070). Since the sample size was small and the measurement of serum E2 levels by RIA may not be sensitive enough for the pre-puberty child or infant, it is difficult to draw any firm conclusions at this time. Future study is necessary to develop the most sensitive assay for measuring serum sex hormones [19].

Phthalates exposure can be from multiple routes that include oral, dermal, or inhalational exposures. In addition to being widely used for industrial purposes, phthalates such as DEHP or DINP are also extensively used in many personal-care and consumer products worldwide [3], [4], [5]. Several studies have shown that one of the main sources of exposure to phthalates in the general population is in the migration from daily-use plastic wrappers or plastic bags when contacted with high temperatures or usage of plastic materials during food production processes [20], [21], [22]. Although this study collected detailed exposure data of phthalates-tainted food items, no information about other routes of phthalates exposure were available which might confound our results. However, in this study, we still found exposure to more than 500 ppm DEHP-tainted foodstuffs, and it is unlikely such a high concentration would be leached from daily-use plastic wrappers or plastic bags, resulting in the decrease of serum TSH levels.

Since DINP is less toxic than DEHP and most foodstuffs were contaminated by DEHP in this incident [1], [7], [8], the information about DINP concentration in affected foods is scant. Thus, we did not examine the relationship between DINP exposure and endocrine profile. AST, ALT, BUN, and creatinine, which represent the functions of the liver and kidney, did not change, even among the highest exposure group. The findings suggest the liver and kidney are not the main targeted organs by DEHP or DINP.

The major limitation of this study is small sample size which can limit our statistical power to detect the significant impact of this scandal on some endocrine dysfunctions. Second, only ∼40% of study children participated in this study due to the main concern of phlebotomy by their parents. In addition, the exposure concentration of DEHP or DINP was not available for all study children. Thus, selection and information biases are likely. Third, T3, instead of free T3, was measured in this study. The free (unbound) portion of free T3 is considered to be more relevant to the biological action than T3. Fourth, the presence of type 1 error is likely, since many tests were performed in the baseline and 6 months follow-up. Fifth, this study was conducted in one medical center; the generalizability of our findings to the whole Taiwan is possibly limited. Finally, the parents were probably aware of the exposure of interest of their children before the questionnaire. Random misclassification of exposure of interest is possible, since the parents of study children did not know the measurement findings of outcome of interest.

In conclusion, our findings add important information regarding the hazards of exposure to phthalates from intentional distribution of phthalate-contaminated foodstuffs in susceptible populations such as children. When establishing TDI recommendations, we should also take groups of susceptible populations into consideration. A long-term follow-up of these affected children concerning their endocrine profiles and growth and neurodevelopment is warranted.

## Supporting Information

Figure S1 **The Spearman correlation between serum and plasma TSH levels by radioimmunoassay (RIA) among the 10 study children.**
(TIF)

Figure S2 **The Spearman correlation of serum TSH levels by radioimmunoassay (RIA) and enzyme immunoassay (EIA) among the 10 study children.**
(TIF)

Table S1 **Characteristics and Clinical Findings of Study Children Categorized by Exposure to Phthalates-tainted Foodstuffs.**
(DOCX)

Table S2 **Differences of Endocrine Hormone Levels in Serum Between 6 Months Follow-up and Baseline Categorized by Exposure to Phthalates-tainted Foodstuffs.**
(DOCX)
