# Supplementary material for: Intake of Phthalate-Tainted Foods Alters Thyroid Functions in Taiwanese Children
Source: PLoS One. 2013 Jan 30;8(1):e55005. doi: 10.1371/journal.pone.0055005 (PMC3559382; doi:10.1371/journal.pone.0055005)
Supplement: Table S1 — Characteristics and Clinical Findings of Study Children Categorized by Exposure to Phthalates-tainted Foodstuffs. (DOCX) [file pone.0055005.s003.docx]

**Table S1. Characteristics and Clinical Findings of Study Children Categorized by Exposure to Phthalates-tainted Foodstuffs.**

| **Variables** | **Exposed group** | **Non-exposed group** |  |
| --- | --- | --- | --- |
|  | **(≥ 1.0 ppm)** | **(< 1.0 ppm)** |  |
|  | **N=52** | **N=8** | **P Value^1^** |
| Mean ± SD (Median, IQR) or N (%) | | | |
| Age (yrs) | 4.9 ± 2.8 | 4.5 ± 2.6 | 0.709 |
|  | (5.0, 2.0-7.0) | (4.0, 3.0-6.0) |  |
| Gender | | | |
| Female | 14 (26.9) | 1 (12.5) | 0.666 |
| Male | 38 (73.1) | 7 (87.5) |  |
| High (cm) | 108.3 ± 21.2 | 106.2 ± 16.3 | 0.916 |
|  | (110.5, 89.0-124.0) | (107.0, 99.0-124.0)^2^ |  |
| Weigh (kg) | 21.5 ± 12.5 | 18.9 ± 6.0 | 0.842 |
|  | (18.0, 12.5-25.0) | (20.0, 14.5-24.0)^2^ |  |
| BMI (Kg/m^2^) | 16.9 ± 3.5 | 16.4 ± 1.6 | 0.925 |
|  | (15.9, 14.9-17.7) | (16.4, 14.8-17.5)^2^ |  |
| Waist circumference (cm) | 52.9 ± 12.1 | 55.0 ± 4.4 | 0.235 |
|  | (51.0, 46.0-58.0) | (54.5, 52.0-57.2)^3^ |  |
| Hip circumference (cm) | 59.1 ± 14.0 | 63.1 ± 5.3 | 0.160 |
|  | (57.5, 50.5-66.8) | (64.0, 60.0-68.0)^3^ |  |
| Endocrine findings | | | |
| **TSH** (**μU/mL**) | **3.07 ± 1.23** | **4.73 ± 1.92** | **0.014** |
|  | **(3.06, 2.34-3.85)** | **(4.74, 3.71-5.70)** |  |
| T4 (μg/dL) | 8.93 ± 2.05 | 8.51 ± 1.97 | 0.520 |
|  | (8.88, 7.63-10.06)^4^ | (7.83, 7.21-11.11)^2^ |  |
| FT4 (ng/dL) | 1.18 ± 0.17 | 1.23 ± 0.26 | 0.722 |
|  | (1.18, 1.09-1.27)^4^ | (1.24, 1.02-1.39)^2^ |  |
| T3 (ng/dL) | 145.5 ± 22.6 | 147.4 ± 22.5 | 0.891 |
|  | (144.5, 127.7-163.3)^4^ | (134.4, 129.8-164.3)^2^ |  |
| E2 (pg/mL) |  |  |  |
| < 8 | 21 (40.4) | 4 (50.0) | 0.708 |
| ≥ 8 | 31 (59.6) | 4 (50.0) |  |
|  |  |  |  |
| TT (ng/dL) |  |  |  |
| < 4 | 35 (68.6)^2^ | 4 (50.0) | 0.425 |
| ≥ 4 | 16 (31.4) | 4 (50.0) |  |
| LH (mIU/mL) |  |  |  |
| < 0.15 | 39 (76.5)^2^ | 8 (100.0) | 0.188 |
| ≥ 0.15 | 12 (23.5) | 0 |  |
| FSH (mIU/mL) | 1.92 ± 1.58 | 1.36 ± 0.89 | 0.453 |
|  | (1.36, 0.90-2.35) | (1.20, 0.74-2.10) |  |
| Biochemical findings | | | |
| AST (IU/L) | 32.0 ± 7.4 | 30.9 ± 4.3 | 0.973 |
|  | (30.0, 27.0-36.0)^2^ | (29.5, 29.0-31.0) |  |
| ALT (IU/L) | 17.7 ± 8.1 | 16.5 ± 2.5 | 0.609 |
|  | (16.0, 13.0-19.0)^2^ | (17.5, 14.0-18.5) |  |
| BUN (mg/dL) | 10.8 ± 3.3 | 12.2 ± 2.2 | 0.161 |
|  | (10.1, 8.6-13.1) | (12.1, 10.1-14.4) |  |
| Creatinine (mg/dL) | 0.36 ± 0.11 | 0.33 ± 0.10 | 0.473 |
|  | (0.36, 0.27-0.43) | (0.32, 0.27-0.39) |  |
| Urinalysis findings | | | |
| Proteinuria (> 1+) | 2 | 0 | - |
| Occult blood (> 1+) | 0 | 0 | - |
| Hematuria (red-cell count, > 5/HPF) | 0 | 0 | - |
| Pyuria (white-cell count, > 5/HPF) | 0 | 0 | - |

Abbreviations: IQR: Interquartile range; BMI: Body mass index; TSH: Thyroid-stimulating hormone; T4: Thyroxine; FT4: Free thyroxine; T3: Triiodothyronine; E2: Estradiol; LH: Luteinizing hormone; FSH: Follicle-stimulating hormone; AST: Aspartate aminotransferase; ALT: Alanine aminotransferase; BUN: Blood urea nitrogen; HPF: High power field.

^1^Mann-Whitney U test for continuous variables and Fischer’s exact test for category variables.

^2^One missing data.

^3^Two missing data.

^4^Eight missing data.
