# Supplementary material for: Intake of Phthalate-Tainted Foods Alters Thyroid Functions in Taiwanese Children
Source: PLoS One. 2013 Jan 30;8(1):e55005. doi: 10.1371/journal.pone.0055005 (PMC3559382; doi:10.1371/journal.pone.0055005)
Supplement: Table S2 — Differences of Endocrine Hormone Levels in Serum Between 6 Months Follow-up and Baseline Categorized by Exposure to Phthalates-tainted Foodstuffs. (DOCX) [file pone.0055005.s004.docx]

**Table S2. Differences of Endocrine Hormone Levels in Serum Between 6 Months Follow-up and Baseline Categorized by Exposure to Phthalates-tainted Foodstuffs.**

| **Differences** | **Exposed group** | | **Non-exposed group** | |
| --- | --- | --- | --- | --- |
| **(Levels at 6 months** | **(≥ 1 ppm)** | | **(< 1 ppm)** | |
| **follow-up − baseline)** |  | |  | |
|  | **N=19** | **P Value^1^** | **N=3** | **P Value^1^** |
| Mean ± SD (Median, IQR) or N (%) | | | | |
| TSH (μU/mL) | 0.22 ± 1.77 | 0.717 | -1.49 ± 0.46 | 0.109 |
|  | (-0.06, -1.24 - 1.06) |  | (-1.72, -1.78 - -0.96) |  |
| T4 (μg/dL) | 0.95 ± 2.73 | 0.170 | 2.97 ± 2.90 | 0.109 |
|  | (1.66, -1.92 - 3.01)^2^ |  | (3.09, 0.02 - 5.81) |  |
| FT4 (ng/dL) | -0.22 ± 0.47 | 0.210 | -0.84 ± 0.62 | 0.109 |
|  | (-0.01, -0.77 - 0.14)^2^ |  | (-1.08, -1.30 - -0.13) |  |
| **T3 (ng/dL)** | **-15.22 ± 24.10** | **0.019** | -15.40 ± 58.26 | 0.593 |
|  | **(-16.39, -33.80 - 0.37)^2^** |  | (-13.24, -74.71 - 41.76) |  |

Abbreviations: IQR: Interquartile range; TSH: Thyroid-stimulating hormone; T4: Thyroxine; FT4: Free thyroxine; T3: Triiodothyronine.

^1^Wilcoxon signed ranks test.

^2^One missing data.
